# Initiation timing and treatment duration of eltrombopag added to immunosuppressive therapy in aplastic anemia: a systematic review and meta-analysis

**DOI:** 10.3389/fonc.2026.1766549

**Published:** 2026-07-22

**Authors:** Shuai Tan, Mingyue Shang, Yixian Guo, Li Su, Huanyuan Wang, Qiang Ma, Wuhan Hui, Jing Ni, Zehao Cai, Huizhen He, Yaofang Cao, Yuxin Li, Yumeng Li, Yaochi Chen, Jing Sun, Wanling Sun

**Affiliations:** 1 Department of Hematology, Xuanwu Hospital, National Center for Neurological Disorders, National Clinical Research Center for Geriatric Diseases, Capital Medical University (XWH-CMU), Beijing, China; 2 Comprehensive Center for Neuro-oncology, Xuanwu Hospital, Capital Medical University (XWH-CMU), Beijing, China

**Keywords:** aplastic anemia (AA), bone marrow failure (BMF), eltrombopag (EPAG), immunosuppressive therapy (IST), thrombopoietin receptor agonists (TPO-RA)

## Abstract

**Background:**

Aplastic anemia (AA) is a bone marrow failure syndrome. Whether the combination of TPO-RAs and IST is superior to IST alone is an ongoing debate. This meta-analysis compares the efficacy of EPAG+IST versus IST alone, and assesses the initiation timing and treatment duration of eltrombopag (EPAG) across different age groups of AA patients.

**Methods:**

The literature was retrieved from Chinese and English databases up to October 1, 2025. The analysis was conducted using RevMan 5.4 software, employing a fixed-effects model to calculate odds ratios (ORs) and 95% confidence intervals (CIs) for the outcomes. The *I²* statistic was used to assess heterogeneity among included studies.

**Results:**

21 studies involving 2,239 patients were included. The overall response rate in the EPAG+IST group was higher at 3 months (OR = 2.13, 95% CI 1.65 – 2.74, P < 0.00001) and 6 months (OR = 2.13, 95% CI 1.73 – 2.61, P < 0.00001). There was no significant difference between the two groups at 12 months (OR = 1.14, 95% CI 0.86 – 1.51, P = 0.36, I² = 35%).

**Conclusion:**

The addition of EPAG to IST may improve early hematological responses at 3 and 6 months in patients with AA, with no significant difference at 12 months. Concurrent initiation (within 7 days) was associated with earlier responses in adults.

**Systematic review registration:**

https://www.crd.york.ac.uk/prospero/, identifier CRD42024604778

## Highlights

- In patients with AA, the addition of EPAG to IST improves hematological responses within 3 to 6 months compared with IST alone, with no significant difference at 12 months.
- In adults, concurrent initiation of EPAG with IST (within 7 days) was associated with improved responses at 3 months, while delayed initiation (>7 days) showed benefit by 6 months.
- In children, no significant added benefit from EPAG was observed at 3 months, with responses emerging by 6 months.

## 1 Introduction

Aplastic anemia (AA), a bone marrow failure (BMF) syndrome, is characterized by marrow hypoplasia and peripheral blood cytopenia (1). The pathophysiology of BMF can arise from chemical or physical damage, a compromised immune system (primarily due to T cells), or constitutional genetic defects affecting immune regulation (2). The incidence of AA ranges from 0.6 to 7 cases per million people per year, with a higher rate observed in adolescents and older adults (3). Frontline treatment of AA depends on disease severity, donor availability, and patient age, primarily involving immunosuppressive therapy (IST) and hematopoietic stem cell transplantation (HSCT). Standard IST, including anti-thymocyte globulin (ATG) and cyclosporine A (CsA), yields a hematologic response rate and transfusion independence of 60 to 70% (4, 5). Nonetheless, about one-third of patients relapse, and 10 to 15% may develop cytogenetic abnormalities in the long term (5).

Eltrombopag (EPAG), an oral thrombopoietin-receptor agonist, is effective in patients with AA refractory to immunosuppression (6, 7). Recent studies indicate that adding EPAG to IST is associated with significantly higher rates of hematologic response in patients with severe AA compared to standard IST (6, 8). However, response rates with EPAG combined with IST vary between adults and children (8, 9), and also depend on different EPAG maintenance durations (10). In clinical practice, there are no conclusive guidelines regarding the optimal timing for initiating and discontinuing EPAG for different populations. Therefore, we conducted this systematic review and meta-analysis to compare the efficacy of EPAG + IST with that of standard IST in patients with AA and to assess the optimal initiation and maintenance timing of EPAG in both adults and children.

## 2 Methods

The recommendations of the Preferred Reporting Items for Systematic Reviews and Meta-Analyses (PRISMA) guidelines (11) were used to formulate the systematic review and meta-analysis eligibility criteria.

### 2.1 Eligibility criteria

Studies were eligible if they met the following criteria (PICOS framework): Population (P): patients diagnosed with AA of any age and severity; Intervention (I): EPAG combined with immunosuppressive therapy (EPAG+IST); Comparator (C): IST alone, including ATG-based regimens or CsA monotherapy; Outcomes (O): overall response and/or complete response at 3, 6, or 12 months; Study design (S): randomized controlled trials, prospective cohort studies, or retrospective cohort studies with a comparator arm. Case reports, single-arm studies without a comparator, conference abstracts, and review articles were excluded.

### 2.2 Search strategy

Eligible studies published before October 1, 2025, were retrieved by two independent authors (S. Tan and MY. Shang) from PubMed, EMBASE, Web of Science, the Cochrane Library, LILACS, CNKI, WANFANG, VIP, and the Chinese Biomedical Literature Database (CBM). The search terms used to retrieve relevant studies included “Aplastic Anemia”, “Eltrombopag” and “immunosuppressive therapy”. The detailed search strategy for each database is provided in **Supplementary Table 1**. Additional studies were identified from the bibliographies of screened articles as appropriate.

### 2.3 Outcomes and study selection

The primary outcomes were overall response (defined as either complete or partial response) and complete response at 3, 6, and 12 months. Complete response was defined according to each included study’s criteria, which generally required an absolute neutrophil count (ANC) ≥ 1.0 × 10 (9)/L, hemoglobin (Hb) ≥ 100 g/L, and a platelet count ≥ 100 × 10 (9)/L. The secondary outcome was partial response (as defined in each included study) at the same time points. Two authors (YX. Guo and L. Su) independently screened the titles and abstracts of all studies and subsequently examined the full texts of eligible studies. In cases of disagreement during the study selection process, consensus was reached through group discussion with a third author (HY. Wang).

### 2.4 Data extraction

Two authors (Q. Ma and WH. Hui) independently collated the baseline characteristics of each included study using a standardized Excel table. The baseline characteristics were as follows: (1) study data, including the first author, publication year, and study design; (2) patient data, encompassing the number of patients, age categories, and gender distribution; (3) grouping information, detailing the initiation time of EPAG and IST programs; and (4) primary and secondary outcomes.

### 2.5 Quality assessment and bias risk assessment

Two authors (J. Ni and ZH. Cai) independently evaluated the quality of observational studies using the Newcastle-Ottawa Scale (NOS). The NOS evaluates three domains—selection, comparability, and outcome assessment—with a maximum total score of 9 stars. Studies scoring ≥ 6 were considered high quality.

For randomized controlled trials (RCTs), the Cochrane Collaboration tool was employed. Assessments were categorized as “low risk of bias,” “unclear risk of bias,” or “high risk of bias” based on seven criteria: Random sequence generation, allocation concealment, blinding of participants and personnel, blinding of outcome assessment, incomplete outcome data, selective reporting, and other biases. Any disagreements in this process were resolved through discussion or adjudication by a third author (HZ. He).

### 2.6 Statistical analysis

All statistical tests were conducted using RevMan 5.4 software. A fixed-effects model was used for the primary analyses to calculate odds ratios (ORs) with 95% confidence intervals (CIs) for both primary and secondary outcomes. Statistical heterogeneity was assessed using the *I²* statistic and Cochran’s Q test. Subgroup analyses were performed based on the initiation time of EPAG (0–7 days vs. > 7 days), age (< 18 years vs. ≥ 18 years), and region (Asia vs. North America), with between-subgroup differences assessed by the chi-square test for interaction. Studies that included both pediatric and adult patients, EPAG initiation timing varied within the cohort, or that originated outside the predefined regional categories (Asia and North America) were excluded from the corresponding subgroup analyses. To assess the robustness of our findings, we conducted sensitivity analyses using the random-effects model (DerSimonian-Laird method) for all primary outcomes, and additional fixed-effects sensitivity analyses for the 6-month outcomes by (1) study design, (2) exclusion of studies with non-standard IST control arms, and (3) disease severity. For safety outcomes, quantitative pooling was performed when comparable two-arm data were available in at least three included studies.

## 3 Results

### 3.1 Outcomes of the search process

**Figure 1:**
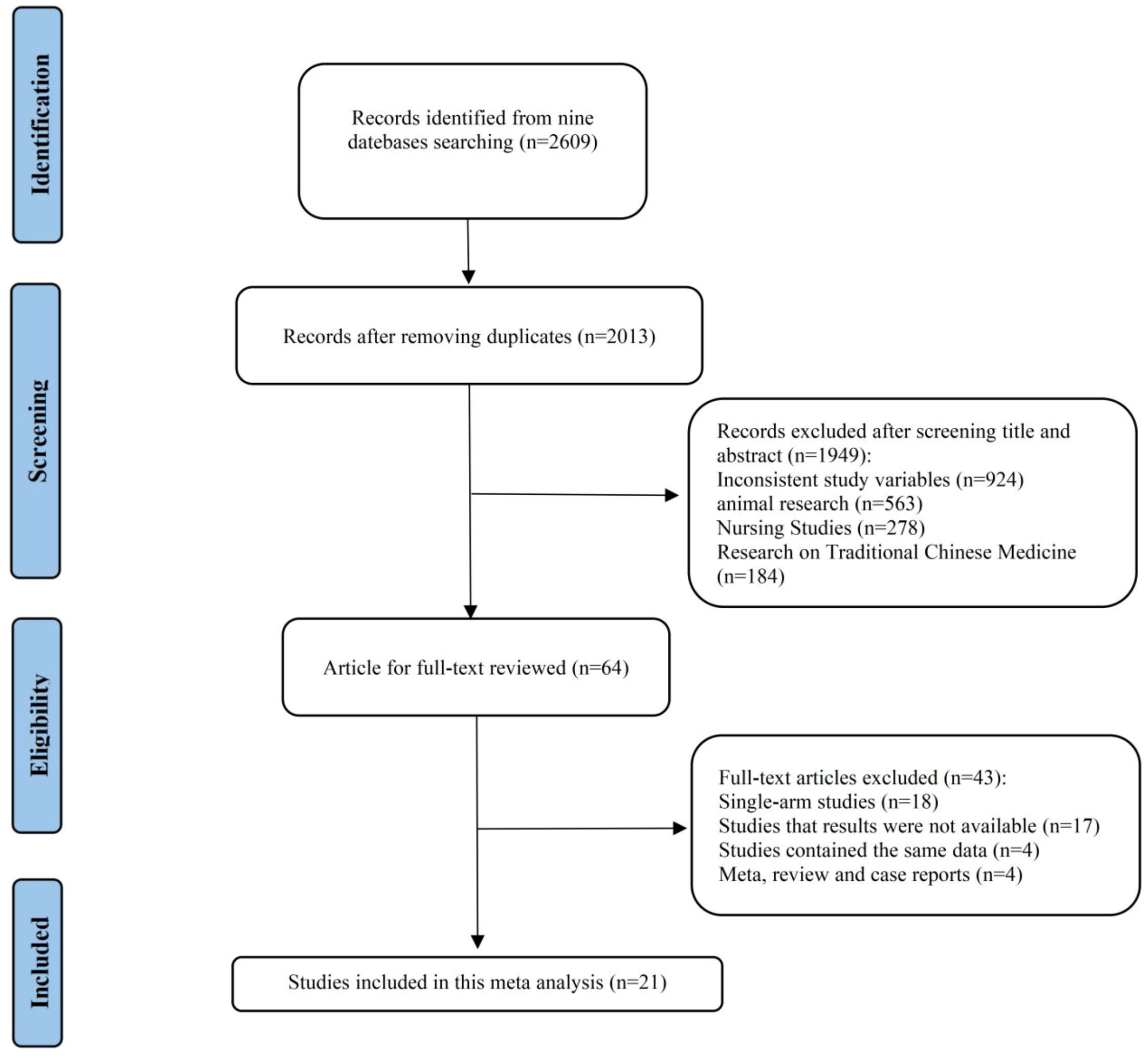
A flow diagram depicting the search process used in this study.

The initial search yielded a total of 2,609 records among all databases, from which 596 duplicates were detected and removed. After reviewing the titles and abstracts of the remaining 2,013 studies according to the exclusion criteria, 1,949 studies were excluded. We then examined the full texts of the remaining 64 articles, resulting in 21 studies that met the inclusion criteria and were ultimately included in our meta-analysis (**Figure 1**).

### 3.2 Study characteristics and quality evaluation

The characteristics of the included studies are summarized in **Table 1**. In total, 2,239 patients were included in our meta-analysis, with 1,059 treated with EPAG + IST and 1,180 treated with IST alone. Of the 21 included studies, 7 were prospective (4 were RCTs) and 14 were retrospective. The quality assessments of the 17 cohort studies are shown in **Table 2**: 6 studies had a NOS score of 9, 5 studies had a score of 8, 4 studies had a score of 7, and 3 studies had a score of 6, all meeting the study criteria. The Cochrane risk of bias assessment for the 4 RCTs indicated low risk of bias and high quality, as presented in Supplemental **Supplementary Figure 1**.

**Table 1:** Characteristics of included studies in the meta-analysis.

Author	Year	Total no.	Study design	Disease severity	Age types of patients/years old	EPAG+IST				IST		
						Patients(n)	Gender (female/male) ,n	EPAG initiation time	Intervention	Patients(n)	Gender (female/male), n	Intervention
Assi (9)	2018	38	Prospective study	Mixed	≥ 18	21	10/11	0-7 days	EPAG+hATG+CsA	17	8/9	hATG+CsA
Cai (32)	2021	86	Retrospective study	Mixed	Mixed	42	22/20	0-7 days	EPAG+CsA	44	23/21	CsA
Chai (33)	2021	35	Retrospective study	SAA	≥ 18	24	9/15	0-7 days	EPAG+rATG+CsA	11	6/5	rATG+CsA
De Latour (8)	2022	197	RCT	SAA/VSAA	Mixed	96	49/52	>7 days	EPAG+hATG+CsA	101	49/52	hATG+CsA
Fang (12)	2021	57	Retrospective study	SAA/VSAA	<18	18	8/10	>7 days	EPAG+pATG+CsA	39	18/21	pATG+CsA
Fang (13)	2023	90	RCT	Mixed	Mixed	45	22/23	0-7 days	EPAG+rATG+CsA	45	22/23	rATG+CsA
Goronkova (19)	2023	98	RCT	SAA/VSAA	<18	49	14/35	0-7 days	EPAG+hATG+CsA	49	19/30	hATG+CsA
Groarke (14)	2021	127	Prospective study	SAA	<18	40	17/23	0-7 days	EPAG+hATG+CsA	87	36/51	hATG+CsA
Hu (15)	2022	111	Retrospective study	SAA/VSAA	Mixed	37	13/24	>7 days	EPAG+r/pATG+CsA	74	40/34	r/pATG+CsA
Jie (16)	2021	42	Retrospective study	Mixed	<18	14	NA	Variable timing	EPAG+rATG+CsA	28	NA	rATG+CsA
Jin (10)	2022	121	Retrospective study	SAA/VSAA	≥ 18	54	26/28	0-7 days	EPAG+rATG+CsA	67	30/37	rATG+CsA
Lesmana (20)	2021	25	Retrospective study	SAA	<18	9	2/7	0-7 days	EPAG+hATG+CsA	16	12/4	hATG+CsA
Liang (34)	2023	89	Retrospective study	Mixed	≥ 18	42	23/19	0-7 days	EPAG+CsA	47	24/23	CsA
Lin (17)	2023	76	Retrospective study	NSAA	≥ 18	45	24/21	0-7 days	EPAG+CsA	31	16/15	CsA
Martynova (35)	2021	21	Retrospective study	Mixed	≥ 18	12	8/4	0-7 days	EPAG+hATG+Tacrolimus	9	6/3	hATG+Tacrolimus
Patel (28)	2022	280	Prospective study	SAA/VSAA	Mixed	178	NA	Variable timing	EPAG+hATG+CsA	102	NA	hATG+CsA
Shinn (27)	2023	82	Retrospective study	SAA/VSAA	≥ 18	48	23/25	0-7 days	EPAG+hATG+CsA	34	16/18	hATG+CsA
Yang (18)	2021	125	RCT	Mixed	≥ 18	63	25/38	0-7 days	EPAG+CsA	62	27/35	CsA
Zaimoku (25)	2021	416	Retrospective study	SAA	≥ 18	176	89/87	Variable timing	EPAG+hATG+CsA	240	99/141	hATG+CsA
Zhang (36)	2022	63	Retrospective study	SAA	<18	31	15/16	0-7 days	EPAG+rATG+CsA	32	16/16	rATG+CsA
Zhao (37)	2023	60	Retrospective study	SAA/VSAA	<18	15	5/10	0-7 days	EPAG+r/pATG+CsA	45	19/26	r/pATG+CsA

EPAG, Eltrombopag, IST: Immunosuppressive therapy; RCT, Randomized controlled trial; NA, no report; SAA, severe aplastic anemia; VSAA, very severe aplastic anemia; NSAA, non-severe aplastic anemia; hATG, horse antithymocyte globulin; rATG, rabbit antithymocyte globulin; pATG, pig antithymocyte globulin; CsA, Cyclosporin A.

**Table 2:** Quality assessment of observational studies included in this meta-analysis.

	Selection				Comparability			Outcome/Exposure		Scores
	Representativ-eness of the exposed cohort	Selection of the non-exposed cohort	Determination of exposure	Ascertainment of no outcome before the study	Matching the most important factors	Control of confounding factors	Evaluation of outcome	Adequacy of follow up	Adequacy of follow up	
Zhao (37)	1	1	1	1	1	1	1	1	1	9
Patel (35)	1	1	1	1	1	0	1	1	1	8
Jin (10)	1	1	1	1	0	1	1	1	1	8
Martynova (35)	1	1	1	1	0	0	1	1	1	7
Fang (13)	1	1	1	1	1	1	1	1	1	9
Lesmana (20)	1	0	1	0	0	1	1	1	1	6
Lin (17)	1	1	1	1	1	1	1	1	1	9
Zhang (36)	1	1	1	1	1	1	1	1	1	9
Zaimoku (25)	1	0	1	0	0	1	1	1	1	6
Jie (16)	1	1	0	1	0	0	1	1	1	6
Groarke (14)	1	0	1	0	1	1	1	1	1	7
Fang (13)	1	1	1	1	1	1	1	1	1	9
Assi (9)	1	1	1	1	0	1	1	1	1	8
Hu (15)	1	0	1	1	1	1	1	1	1	8
Chai (33)	1	1	1	1	0	0	1	1	1	7
Liang (34)	1	1	1	1	0	1	1	1	1	8
Cai (32)	1	0	1	1	1	1	1	0	1	7
Shinn (27)	1	1	1	1	1	1	1	1	1	9

The Newcastle-Ottawa Scale (NOS) was used to evaluate the quality of the observational studies. Scores ranged from 0 to 9: scores of 1 or less were considered low quality (high risk of bias), scores of 2–5 were medium quality (unclear), and scores of 6–9 were high-quality literature (low risk of bias).

### 3.3 Outcomes of this meta−analysis

#### 3.3.1 Overall response

**Figure 2:**
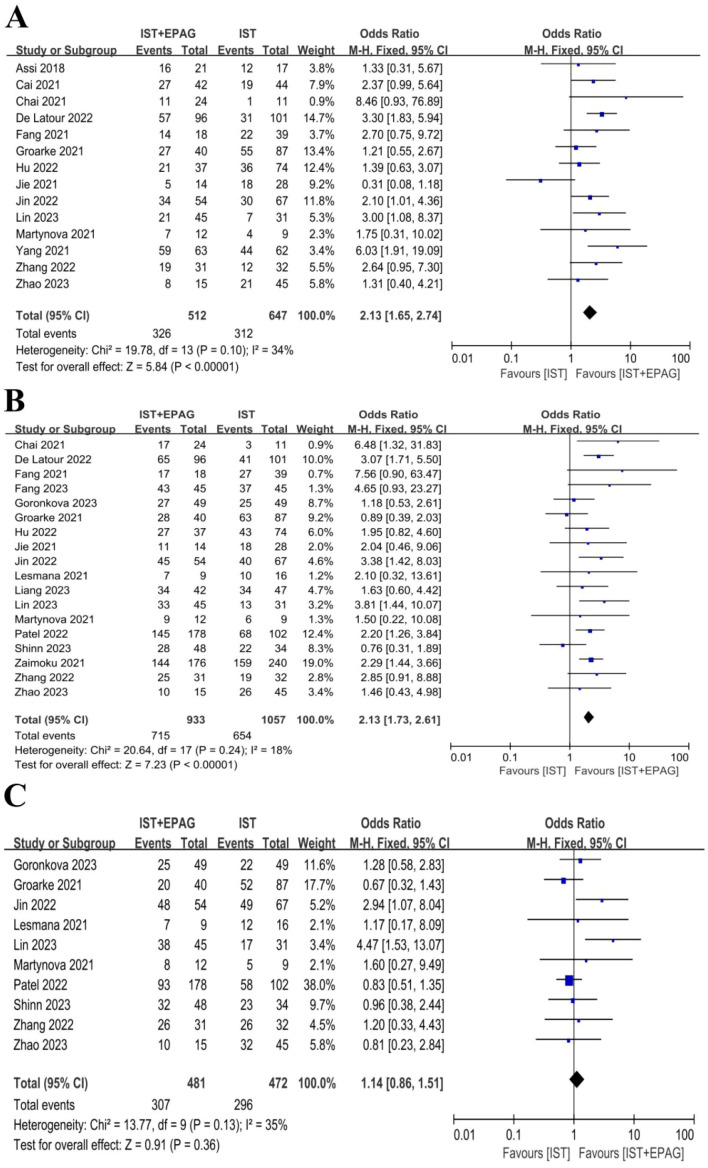
Overall response at 3, 6, and 12 months. **(A)** Overall response at 3 months. **(B)** Overall response at 6 months. **(C)** Overall response at 12 months. EPAG, Eltrombopag; IST, Immunosuppressive therapy; CI, Confidence interval.

The overall response after 3 months was evaluated in 14 studies, encompassing 1,159 patients with AA (**Figure 2A**). The results indicated that the overall response rate in the EPAG combined with IST group was higher than in the IST group (OR=2.13, 95% CI 1.65–2.74, P < 0.00001), with low heterogeneity among these studies (P = 0.10, *I²* = 34%). At 6 months, 18 studies including 1,990 patients with AA were evaluated (**Figure 2B**), showing a similar trend: the overall response rate in the EPAG combined with IST group was higher than in the IST group (OR=2.13, 95% CI 1.73–2.61, P < 0.00001), again with low heterogeneity (P = 0.24, *I²* = 18%). At 12 months, 10 studies with 953 patients were assessed (**Figure 2C**), revealing no significant difference between the EPAG + IST and IST alone groups according to the pooled results (OR=1.14, 95% CI 0.86–1.51, P = 0.36, *I²* = 35%).

#### 3.3.2 Complete response

**Figure 3:**
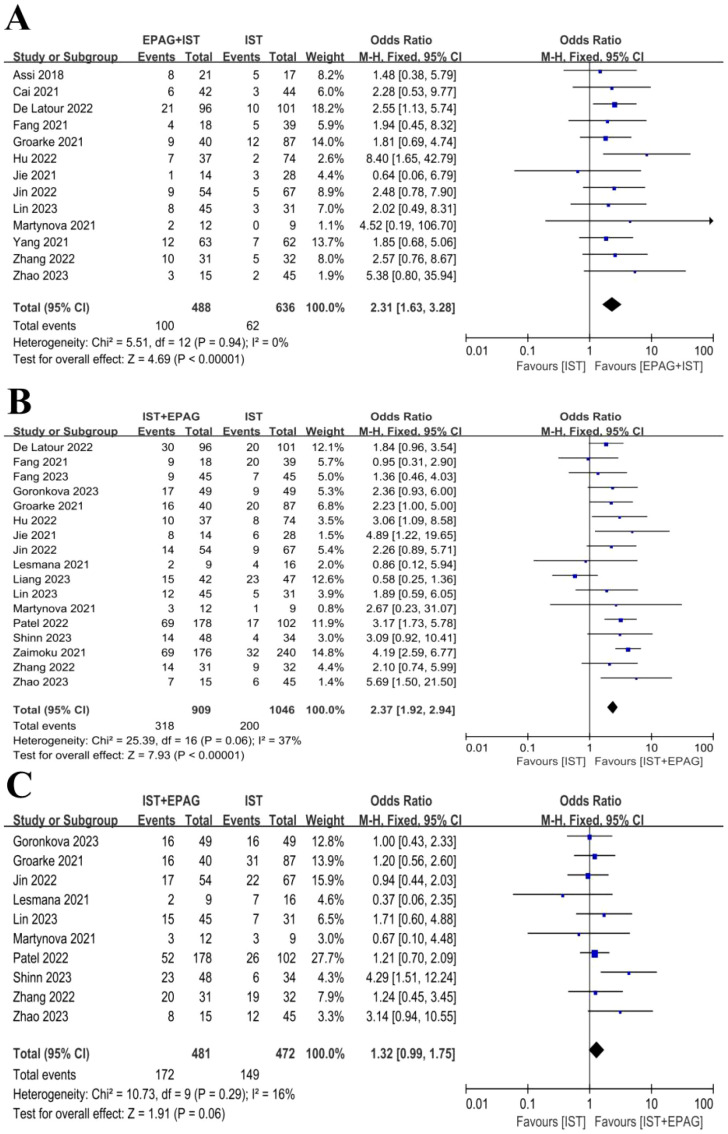
Complete response at 3, 6, and 12 months. **(A)** Complete response at 3 months. **(B)** Complete response at 6 months. **(C)** Complete response at 12 months. EPAG, Eltrombopag; IST, Immunosuppressive therapy; CI, Confidence interval.

The complete response at 3 months was evaluated in 13 studies involving 1,124 patients with AA (**Figure 3A**). Our analysis suggested that the complete response rate in the EPAG combined with IST group was higher than in the IST group (OR=2.31, 95% CI 1.63–3.28, P < 0.00001), with no heterogeneity (P = 0.94, *I²* = 0%). At 6 months, 17 studies including 1,955 patients were assessed (**Figure 3B**), indicating a higher complete response rate in the EPAG combined with IST group (OR=2.37, 95% CI 1.92–2.94, P < 0.00001), with some heterogeneity present (P = 0.06, *I²* = 37%). At 12 months, 10 studies with 953 patients were analyzed (**Figure 3C**), finding no significant difference between the EPAG + IST and IST alone groups (OR=1.32, 95% CI 0.99–1.75, P = 0.06, *I²* = 16%).

#### 3.3.3 Subgroup analysis

**Figure 4:**
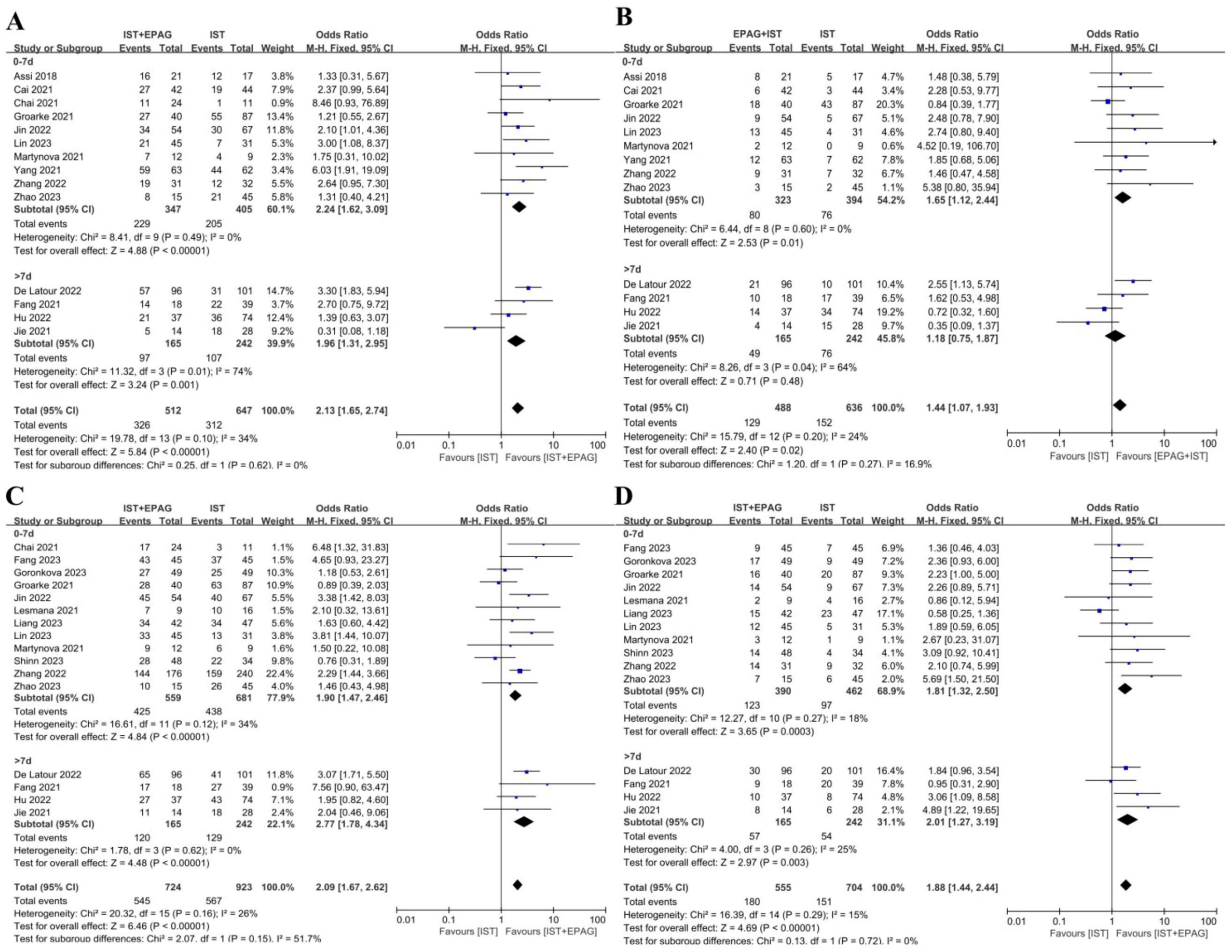
Subgroup analysis of EPAG initiation time at 3 and 6 months. **(A)** Overall response at 3 months. **(B)** Complete response at 3 months. **(C)** Overall response at 6 months. **(D)** Complete response at 6 months. EPAG, Eltrombopag; IST, Immunosuppressive therapy; CI, Confidence interval.

Subgroup analysis based on EPAG initiation time was performed using overall response and complete response after 3 and 6 months as indices (**Figure 4**). The start of IST treatment was designated as day 0, with subgroup analyses separating EPAG initiation at day 7 after IST treatment as the cut-off. The results for the 0-7 days EPAG initiation subgroup indicated that IST combined with EPAG improved both overall and complete responses at 3 and 6 months for AA patients. Conversely, the >7 days EPAG initiation subgroup showed improvements in overall and complete responses only at 6 months. Overall, the P-values for interactions were greater than 0.05, indicating that the overall and complete responses were not significantly influenced by the timing of EPAG initiation in AA patients.

#### 3.3.4 Subgroup analysis of different ages

**Figure 5:**
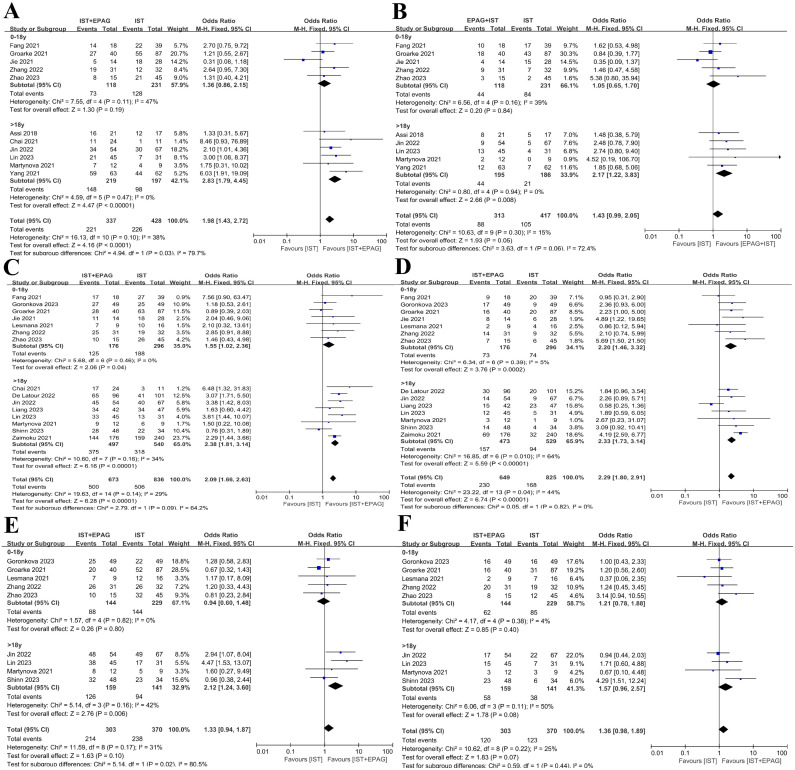
Subgroup analysis of different ages at 3, 6, and 12 months. **(A)** Overall response at 3 months. **(B)** Complete response at 3 months. **(C)** Overall response at 6 months. **(D)** Complete response at 6 months. **(E)** Overall response at 12 months. **(F)** Complete response at 12 months. EPAG, Eltrombopag; IST, Immunosuppressive therapy; CI, Confidence interval.

Subgroup analysis was performed using overall response and complete response at 3, 6, and 12 months as indices, categorized by age (**Figure 5**). The results for the subgroup of patients aged <18 years (children) indicated that IST combined with EPAG improved overall and complete response at 6 months. However, there was no effect at 3 and 12 months. For the subgroup of patients aged ≥18 years (adults), IST combined with EPAG improved overall response and complete response at 3 and 6 months. Additionally, it affected overall response at 12 months but did not influence complete response at that time. Overall, the P-value for the interaction of overall response at 3 months was less than 0.05, indicating that the age of AA patients had a significant effect on this response.

#### 3.3.5 Subgroup analysis of different regions

**Figure 6:**
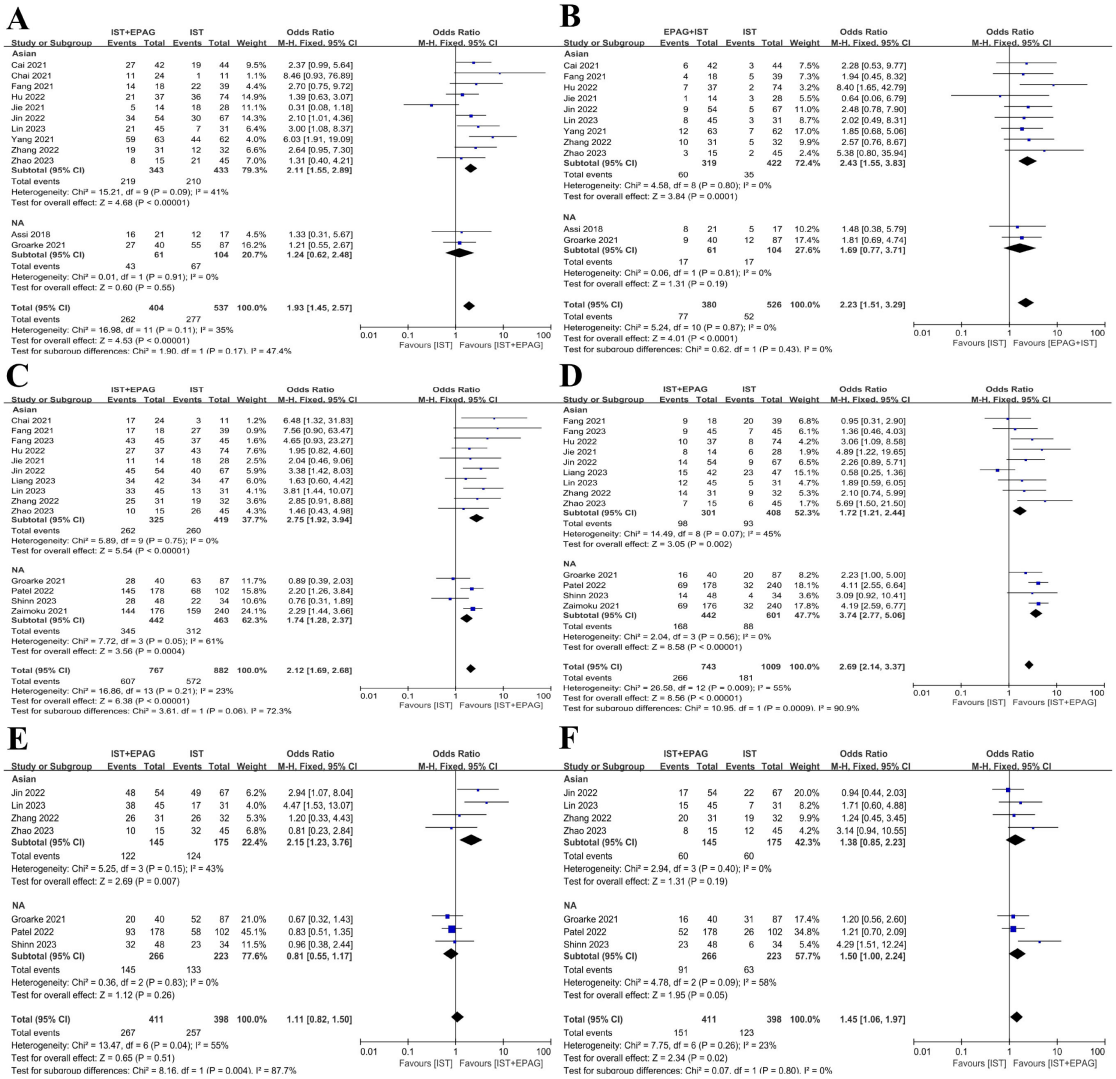
Subgroup analysis of different regions at 3, 6, and 12 months. **(A)** Overall response at 3 months. **(B)** Complete response at 3 months. **(C)** Overall response at 6 months. **(D)** Complete response at 6 months. **(E)** Overall response at 12 months. **(F)** Complete response at 12 months. EPAG, Eltrombopag; IST, Immunosuppressive therapy; CI, Confidence interval; NA, North America.

Using overall response and complete response at 3, 6, and 12 months as indices, subgroup analysis was performed by region (**Figure 6**). The analysis for the Asian region revealed that IST combined with EPAG improved overall response and complete response at 3 and 6 months, but had no effect at 12 months. In contrast, the North America (NA) region analysis indicated that IST combined with EPAG had no effect except on complete response after 6 months.

#### 3.3.6 Sensitivity analyses

To assess the robustness of our primary findings, we performed multiple sensitivity analyses focused on the 6-month outcomes, which represent the most clinically relevant time point with the largest pooled sample size.

First, random-effects analyses for all primary outcomes yielded pooled estimates highly consistent with the fixed-effects analyses, preserving both direction and statistical significance (**Supplementary Figures 2**, **S3**). Second, stratification by study design (3 RCTs versus 15 observational studies) showed consistent effects of EPAG+IST in both subgroups for both overall and complete responses, with no significant between-subgroup differences (test for subgroup differences: P = 0.63 and P = 0.26, respectively; **Supplementary Figure 4**). Yang 2021 did not report 6-month outcomes and was excluded from this analysis. Third, after excluding the two studies (Liang 2023 and Lin 2023) with CsA monotherapy control arms that reported 6-month data, the pooled estimates remained essentially unchanged (6-month ORR: OR = 2.10, 95% CI 1.69–2.60; 6-month CRR: OR = 2.66, 95% CI 2.12–3.34; **Supplementary Figure 5**), indicating that the observed benefit was not driven by indirectness bias from non-standard control regimens. Fourth, stratification by disease severity (12 SAA/VSAA studies versus 6 mixed/NSAA studies) showed consistent effects on overall response (test for subgroup differences: P = 0.22), but the complete response benefit was more pronounced in the SAA/VSAA subgroup (OR = 2.74) than in the mixed/NSAA subgroup (OR = 1.48; test for subgroup differences: P = 0.02; **Supplementary Figure 6**), suggesting that the CRR benefit of EPAG + IST may be more clearly demonstrated in more severe disease.

### 3.4 Publication bias

Stata 15 was used to evaluate publication bias in overall response and complete response at the 3, 6, and 12 months intervals. Begg’s and Egger’s tests were conducted, revealing no significant publication bias in this study (3 months ORR: Begg’s test P = 0.511, Egger’s test P = 0.669; 3 months CRR: Begg’s test P = 0.502, Egger’s test P = 0.697; 6 months ORR: Begg’s test P = 0.596, Egger’s test P = 0.479; 6 months CRR: Begg’s test P = 0.837, Egger’s test P = 0.879; 12 months ORR: Begg’s test P = 0.592, Egger’s test P = 0.797; 12 months CRR: Begg’s test P = 1.000, Egger’s test P = 0.854).

### 3.5 Adverse events

The most common adverse event related to EPAG is liver dysfunction, primarily characterized by increased bilirubin, aspartate aminotransferase, and alanine transaminase levels (8, 10, 12–18). Liver dysfunction led to treatment interruptions (7, 19) or dose reductions (20) in some study patients. Generally, liver function abnormalities in most AA patients were temporary and reversible; however, 10 out of 49 (20%) pediatric patients in Goronkova et al.’s study (19) experienced grade 3 to 4 liver function abnormalities that persisted for more than 21 days after drug discontinuation. Among the four studies that reported comparable two-arm data on any-grade hepatic dysfunction (de Latour 2022, Goronkova 2023, Jin 2022, and Lesmana 2021; 255 patients in the EPAG + IST group and 285 in the IST alone group), the pooled analysis showed no significant increase in hepatic dysfunction with the addition of EPAG (OR = 0.71, 95% CI 0.48–1.03, P = 0.07, *I²* = 8%; **Supplementary Figure 7**). In Lesmana et al.’s study, renal insufficiency was more prevalent among patients receiving EPAG. Additionally, gastrointestinal adverse reactions, primarily abdominal distension and mild diarrhea, were noted in Lin et al.’s study (17). None of these adverse events resulted in dose reduction or discontinuation of EPAG. Other reported adverse events included febrile neutropenia, infection, hypertension, skin hyperpigmentation, rash, myalgia, joint pain, and cardiac disorders. Quantitative pooling of other safety outcomes was not feasible due to inconsistent reporting or insufficient two-arm data across the included studies.

## 4 Discussion

Many studies have confirmed the efficacy of EPAG + IST versus IST alone for treating AA; however, a unanimous conclusion is still lacking. De Latour et al.’s multicenter trial (8) showed that complete response at 3 months was 10% in the IST combined with EPAG group and 22% in the IST group (odds ratio, 3.2; 95% confidence interval [CI], 1.3 to 7.8; P = 0.01). There was no significant difference in overall response or complete response at 6 months. Conversely, Jin et al.’s study (10) demonstrated that the overall response rates (ORR) at 3, 6, and 12 months were higher in the IST combined with EPAG group (P = 0.002, 0.028, 0.006, 0.031), while the complete response rates (CRR) were similar between the two groups. Our meta-analysis indicated that overall response and complete response at 3 and 6 months improved in the IST combined with EPAG group compared to the IST group, with no significant differences at 12 months. Importantly, the robustness of our primary findings is supported by the consistency between fixed-effects and random-effects analyses (**Supplementary Figures 2**, **S3**). This suggests that adding EPAG can increase the rate, rapidity, and strength of hematologic responses in AA patients, with a desirable therapeutic effect sustained during 6 months of treatment.

Regarding clinical treatment, it remains to be determined when EPAG should be initiated after basic IST therapy for optimal results. The phase 2 NIH trial (6) suggested that the best outcomes were achieved when EPAG was introduced on day 0. To prevent possible cumulative toxicity from concurrent ATG administration, EPAG was initiated on day 14 in de Latour et al.’s trial (12), which found no detrimental effect on early hematologic response. Our subgroup analysis based on EPAG initiation time showed that concurrent therapy (adding EPAG within 7 days of IST treatment) significantly improved overall and complete responses at both 3 and 6 months, while delayed initiation (>7 days) showed significant improvements only at 6 months. Given that survival and long-term outcomes for AA patients correlate with the quality of hematologic response at 3 months and the presence of early recovery after ATG administration (21), our analysis suggests that concurrent therapy may be associated with a more favorable long-term prognosis for AA patients.

We also conducted a subgroup analysis based on age to assess whether age affected the efficacy of EPAG. Results indicated that EPAG + IST was more effective than IST alone in adults; however, in pediatric subgroup, the addition of EPAG did not demonstrate significant efficacy overall. Age is known to be a prognostic factor in AA patients treated with IST, particularly with antithymocyte globulin combined with cyclosporine being effective in children (21–24). We speculate that this discrepancy may be due to pediatric patients responding better to IST treatment than adults, resulting in a less significant effect of EPAG in the younger subgroup. Additionally, adult patients with AA may have a higher ANC than pediatric patients, and some studies suggest that EPAG is more effective in AA patients with higher ANC (25, 26). Although current guidelines do not include EPAG combined with standard IST therapy as a first-line treatment option for pediatric SAA, it has been demonstrated that EPAG not only increases ANC and CR rates in pediatric patients, but also that the early addition of EPAG can help rapidly identify nonresponders who are in urgent need of HSCT. The varying efficacy of EPAG among different age groups warrants further investigation in prospective stratified studies.

Subgroup analysis by region revealed that in the Asian subgroup, the addition of EPAG to IST significantly improved ORR and CRR at 3 and 6 months. In contrast, the North America (NA) subgroup showed no effect on overall response, except for complete response at 6 months. This discrepancy may stem from different indications for EPAG in various regions and patient age demographics. Currently, EPAG is approved only in specific countries to treat AA, with most patients in the NA subgroup having severe AA (SAA) or very severe AA (VSAA) (25, 27, 28). Additionally, two NA subgroup articles (14, 28) included pediatric patients, and the limited number of studies may have influenced the results of this analysis.

Sensitivity analyses further supported the robustness of our primary findings. The consistency of effects between RCT and observational study subgroups (**Supplementary Figure 4**) validated the pooling of studies with different designs, and the exclusion of studies with non-standard IST regimens (CsA monotherapy) yielded pooled estimates consistent with the primary analyses (**Supplementary Figure 5**), indicating that the observed benefit of EPAG+IST is not attributable to indirectness bias from heterogeneous control arms.

Sensitivity analysis by disease severity showed that while the effect on 6-month overall response was consistent across SAA/VSAA studies and mixed/NSAA studies, the complete response benefit was more pronounced in studies restricted to SAA/VSAA patients (**Supplementary Figure 6**). This pattern may reflect the greater residual hematopoietic capacity and favorable IST response of NSAA patients, leaving less room for additional benefit from EPAG, and may also be influenced by the higher proportion of non-standard control regimens within the mixed/NSAA subgroup.

Our quantitative analysis of any-grade hepatic dysfunction across four studies with comparable two-arm data showed no significant increase in liver function abnormalities with the addition of EPAG, supporting the overall hepatic safety of EPAG when combined with IST, although careful monitoring of transaminase and bilirubin levels remains essential. Beyond liver function abnormalities, the risk of clonal evolution from EPAG treatment cannot be overlooked. Current studies suggest that alterations in the immune bone marrow environment may contribute to the clonal evolution of AA patients (29), though the addition of EPAG has not significantly increased the risk of high-risk clonal evolution events (6, 30). Groarke et al. (30) evaluated predictors of clonal evolution in AA patients post-IST treatment, finding that pretreatment age > 48 years and ANC > 0.87 × 10 (9)/L were strong predictors of high-risk evolution. Moreover, RUNX1, splicing factor, and ASXL1 somatic mutations detected 6 months after IST treatment could predict high-risk evolution. In future IST or EPAG treatments, it is crucial to consider the characteristics of clonal evolution in AA patients and extend follow-up for targeted monitoring.

The strategy for tapering and discontinuation of EPAG also merits explicit consideration. First, the absence of incremental benefit beyond 12 months in our analysis suggests that prolonged exposure beyond one year is unlikely to yield further hematological gains once a sustained response has been achieved. Second, this is consistent with reports from the NIH cohorts, in which EPAG was successfully tapered and discontinued in patients with robust trilineage recovery (7), and in which relapses tended to cluster around cyclosporine withdrawal rather than EPAG cessation (28) — arguing against EPAG dependence as a major driver of relapse in deep responders. Third, theoretical risks associated with prolonged TPO-RA exposure — including persistent liver dysfunction (8, 10, 12–14), iron depletion as recently reported by Young et al. (31), and the earlier onset of clonal evolution (30) — further support minimizing unnecessary drug exposure. Collectively, these observations support a response-adapted approach in principle: continuing EPAG until a robust hematological response is consolidated, followed by gradual tapering and eventual discontinuation under structured monitoring for relapse and late clonal events.

Our meta-analysis has some limitations. First, among the 21 studies included, there were 17 cohort studies and 4 RCTs; therefore, the mixed results from different study types may affect the conclusions. Second, certain baseline characteristics, such as disease severity, were not consistently reported across included studies, potentially leading to bias. Our sensitivity analysis by severity suggested a more pronounced CRR benefit in SAA/VSAA studies than in mixed/NSAA studies, which should be interpreted cautiously given the relatively small mixed/NSAA subgroup. Third, definitions of complete response and partial response varied by region due to ethnic heterogeneity, which may also introduce potential bias. For example, the definition of complete response in Zhao et al.’s Asian study was an ANC ≥1.5×10 (9)/L, while in De Latour et al.’s European study, it was an ANC ≥1×10 (9)/L. Fourth, the included studies were heterogeneous with respect to EPAG maintenance and tapering protocols, which precluded a formal quantitative comparison of different tapering strategies. The absence of head-to-head data comparing fixed-duration with response-adapted regimens represents an important gap that should be addressed by future prospective trials.

## 5 Conclusion

Our results suggested that the combination of EPAG and IST may improve hematological responses in patients with AA compared to those treated with IST alone. The concurrent therapy of EPAG and IST and maintaining EPAG therapy for 3 to 6 months were associated with significant improvements in hematological responses. In the adult group, the hematologic responses of concurrent therapy and prolonged maintenance (≥ 3 months) were observed earlier, while in the pediatric group, responses tended to emerge later (EPAG response time ≥ 6 months). When EPAG was maintained for more than 12 months, no significant difference in outcomes was observed between the two age groups. The main adverse event associated with EPAG was liver dysfunction, indicating the importance of monitoring indicators such as transaminase and bilirubin levels.

## Data Availability

The original contributions presented in the study are included in the article/**Supplementary Material**. Further inquiries can be directed to the corresponding authors.
