# Supplementary material for: Initiation timing and treatment duration of eltrombopag added to immunosuppressive therapy in aplastic anemia: a systematic review and meta-analysis
Source: Front Oncol. 2026 Jul 22;16:1766549. doi: 10.3389/fonc.2026.1766549 (PMC13437481; doi:10.3389/fonc.2026.1766549)
Supplement: Supplementary file 1 [file DataSheet1.docx]

**Supplemental materials**

**Supplementary Table S1.** Detailed PubMed search strategy.

| **Concept** | **Search terms** |
| --- | --- |
| **#1 Immunosuppressive therapy** | "Immunosuppressive Agents"[MeSH] OR "Immunosuppression Therapy"[MeSH] OR immunosuppressive therapy[Title/Abstract] OR immunosuppressive therapies[Title/Abstract] OR immunosuppressant*[Title/Abstract] OR immunosuppression*[Title/Abstract] OR anti-rejection therap*[Title/Abstract] OR antirejection therap*[Title/Abstract] |
| **#2 TPO-receptor agonists** | Thrombopoietin receptor agonist[Supplementary Concept] OR "eltrombopag"[Supplementary Concept] OR eltrombopag[Title/Abstract] OR Promacta[Title/Abstract] OR Revolade[Title/Abstract] OR Eltrombopag olamine[Title/Abstract] OR "romiplostim"[Supplementary Concept] OR AMG531[Title/Abstract] OR Nplate[Title/Abstract] OR "avatrombopag"[Supplementary Concept] OR Doptelet[Title/Abstract] OR AKR501[Title/Abstract] OR YM477[Title/Abstract] |
| **#3 Aplastic anemia** | "Anemia, Aplastic"[MeSH] OR aplastic anemia*[Title/Abstract] OR aplastic anaemia*[Title/Abstract] OR hypoplastic anemia*[Title/Abstract] |
| **Combination** | (#1 OR #2) AND #3 |
| **Filters** | Published before October 1, 2025; Languages: English, Chinese |

**Supplemental Figure S1.** Quality assessment of RCTs included in this meta-analysis. (A) Risk of bias graph: Judgments about each risk of bias item presented as percentages across all included studies. (B) Risk of bias summary: Judgments about each risk of bias item for each included study.

**Supplementary Figure S2.** Random-effects analysis of overall response at 3, 6, and 12 months. (A) Overall response at 3 months. (B) Overall response at 6 months. (C) Overall response at 12 months. EPAG: eltrombopag, IST: immunosuppressive therapy, CI: confidence interval.

**Supplementary Figure S3.** Random-effects analysis of complete response at 3, 6, and 12 months. (A) Complete response at 3 months. (B) Complete response at 6 months. (C) Complete response at 12 months. EPAG: eltrombopag, IST: immunosuppressive therapy, CI: confidence interval.

**Supplementary Figure S4.** Sensitivity analysis stratified by study design for 6-month outcomes. (A) Overall response at 6 months. (B) Complete response at 6 months. Studies were stratified into randomized controlled trial (RCT) and observational study subgroups. EPAG: eltrombopag, IST: immunosuppressive therapy, CI: confidence interval.

**Supplementary Figure S5.** Sensitivity analysis excluding studies with CsA monotherapy control arms for 6-month outcomes. (A) Overall response at 6 months. (B) Complete response at 6 months. Two studies with CsA monotherapy controls reporting 6-month data (Liang 2023 and Lin 2023) were excluded from this analysis. EPAG: eltrombopag, IST: immunosuppressive therapy, CI: confidence interval.

**Supplementary Figure S6.** Sensitivity analysis stratified by disease severity for 6-month outcomes. (A) Overall response at 6 months. (B) Complete response at 6 months. Studies were stratified into two subgroups including SAA/VSAA and mixed/NSAA patients. EPAG: eltrombopag, IST: immunosuppressive therapy, CI: confidence interval.

**Supplementary Figure S7.** Quantitative analysis of any-grade hepatic dysfunction. EPAG: eltrombopag, IST: immunosuppressive therapy, CI: confidence interval.
